# Profile and risk factors of blood donors who experienced adverse reactions: a cross-sectional study on donor hemovigilance data reported to the national network from 2020 to 2022 in China

**DOI:** 10.3389/fpubh.2025.1567370

**Published:** 2025-11-26

**Authors:** Junhong Yang, Wenqin Zhu, Dongfu Xie, Qing Xu, Xiaojie Guo, Jia Zeng, Yongzhu Xu, Xia Huang

**Affiliations:** 1 Chongqing Blood Center, Chongqing, China; 2 Working Party on Hemovigilance of the Chinese Society of Blood Transfusion, Chongqing, China; 3 Fujian Blood Center, Fuzhou, China; 4 Shanghai Red Cross Blood Center, Shanghai, China; 5 Anhui Blood Center, Hefei, China

**Keywords:** adverse reaction, donor hemovigilance, syncope, vasovagal reaction, risk factor

## Abstract

**Background:**

While international cooperation in blood donor safety surveillance is growing, data from China’s national network remains unpublished. We have established a national donor hemovigilance system to collect data and analyze the current state of blood donor safety in China.

**Materials and methods:**

We conducted a cross-sectional study on donor hemovigilance data reported to the national network from 2020 to 2022 in China. Demographic information and data on adverse donor reactions (ADRs) among blood donors were collected from 85 Blood Services. R software (version 4.2.3) was used for all statistical analyses. Frequency and composition rates were used to describe the data of total donation, as well as data on the different types of ADRs. The Chi-square test was used to analyze risk factors for Vasovagal reactions (VVRs) and inter-group comparisons of VVRs stratified by age (18–22; 23–29; 30–39; 40–49; 50–60), gender (female/male), donation history (first time/repeat), and blood donation volume (200 mL/300 mL/400 mL), type of collection site (fixed site/blood collection shelter/blood collection vehicle/others) and donor source (individual/social group/high school). Lastly, risk factors for blood donation-related syncope were explored.

**Results:**

Between 2020 and 2022, 32,523 cases of ADRs were reported, among which VVRs accounted for 96.8% of all reactions. The overall reported incidence of ADRs was 12.577%. Notably, the incidence of VVRs decreased with increasing donor age. Additionally, the estimated incidences of VVRs were higher among female donors (13.600%) and first-time donors (18.536%) compared to male donors (11.205%) or repeat donors (5.819%). Moreover, 1,120 VVRs with syncope were recorded, accounting for 3.56% of total VVR cases. Finally, donors from outdoor blood collection sites (0.590%) and groups (1.073%) were at a significantly higher risk of syncope compared to those from indoor blood donation sites (0.438%) and individual donors (0.174%).

**Conclusion:**

The reported incidence rate of ADRs related to blood donation was very low. The higher prevalence of VVR and syncope in young, females, first-time donors, college students, and donating in mobile vehicles.

## Introduction

Recently, the reporting, treatment, and prevention of adverse donor reactions (ADRs) have garnered extensive attention (1, 2). These activities form the core of donor hemovigilance (DHV), which refers to the systematic surveillance of ADRs and incidents throughout the whole blood donation process to enhance quality and safety for blood donors (3). Blood donation is a generally safe procedure well-accepted by donors. Nevertheless, a minority of donors may experience undesired ADRs of variable severity during or following blood donation (4). While the majority of blood donors rapidly recover from ADRs with supportive management, even experienced donors are less likely to return following mild complications (5). In recent years, numerous studies have identified risk factors of adverse reactions related blood donation and demonstrated the effectiveness of intervention measures (1, 5, 6).

The International Hemovigilance Network (IHN) developed a system for the annual collection of national aggregate hemovigilance data in 2006 (7), with over 30 national hemovigilance systems globally participating in the program and 24 having reported ADR data to IHN by 2016. However, the latest IHN publication did not include data on ADRs in China (8). In 2021, IHN published a large dataset of ADRs reports over 11 years (2006–2016), covering 138 countries and 155 million blood donors. The overall reported incidence rate of ADRs was 6%, with a median of 3.2% (IQR 1. 1 ~ 10. 1). The average incidence rate of DRVR was 4.6%, with a median incidence rate of 3.1% (IQR 0.6–7.7). The median incidence rates of adverse reactions related to venipuncture (hematoma) were 0.39% (IQR 0.31–1.2) for whole blood donation and 4.2% (IQR 0.69–5.6) for apheresis platelet donation. A recent meta-analysis documented that the incidence of ADRs during or after whole blood donation was 13.6% in China (9). Despite several studies investigating ADRs in the Chinese population, large-scale surveys remain limited.

To compare and benchmark ADR rates across Blood Services and regions, the Chinese Society of Blood Transfusion (CSBT) established a national hemovigilance working party in 2017 and implemented nationwide ADR surveillance in China. A previous article detailed the establishment and development of a national blood donor hemovigilance system in China (10). Between 1 January 2020 and 31 December 2022, formal ADR data collection was conducted using a web-based management system. In this paper, the collected ADR data were analyzed to characterize the profile of blood donors who experienced ADRs in China and promote data utilization and visibility across China.

## Materials and methods

### Study population

All blood donors from 85 Blood Services between 1 January 2020 and 31 December 2022 were included in the study. These accounted for 18.8% of all Blood Services in China. From 2020 to 2022, more than 8.5 million blood donors from the Blood Services participating in this study, accounting for 17.8% of the total number of all blood donors in China during this period. Donors met the standard blood donor eligibility for whole blood and component donations established by the China Ministry of Health (11). Healthy individuals aged 18–55 years are eligible for blood donation for the first time, and repeat donors who met the blood donor criteria with no previous ADRs are eligible up to the age of 60 years. The minimum body weight and hemoglobin level were 50 kg and ≥120 g/L for male donors and 45 kg and ≥115 g/L for female donors, respectively. The minimum interval between two whole blood donations was 6 months, and 14 days for apheresis platelets. After registration and physical examination pre-donation, donors received counseling from professional staff and could choose to donate 200 mL, 300 mL, or 400 mL for whole blood and a single or double treatment dose for apheresis platelets. This study was approved by the Ethics Committee of Chongqing Blood Center (No. 2021-04-26).

### ADR classification criteria and definition

The definition and imputability for ADRs used in this study was proposed by the Working Group on Donor Vigilance of the International Society of Blood Transfusion (ISBT) Working Party on Hemovigilance, in collaboration with IHN and the AABB Donor Hemovigilance Working Group in 2014 (12). Primary ADRs were classified into four major types: complications with predominantly local symptoms, generalized symptoms (vasovagal reactions, VVRs), complications related to apheresis, and other complications related to blood donation. To enhance specificity, the four major ADR categories were subdivided into additional subcategories. We include and exclude the received data based on this criterion.

### ADR and denominator data collection

For consistency, a standardized data collection form, the ADRs report form, was designed and issued to Blood Services for collecting ADR data. For each reaction, donor demographic information (age, gender, weight, height, collection site, and donation history), signs and symptoms, interventions, follow-up information, and reaction type were collected. All reactions, irrespective of severity, were recorded and reported to CSBT. Multiple types of ADRs in a single blood donor were individually recorded and reported. The collected information was registered and audited in a dedicated database. A specific online system was designed for data entry, submission, analysis, and storage. Blood Services reported the collected ADR data monthly via a dedicated web platform^1^ by liaison officers (10). Denominator data were collected using a uniform data collection form from early 2021 to 2023. Donations were classified by age group, donation history, gender, blood donation volume, and type of blood donation site were collected.

### Statistical analysis

All ADR datasets were subjected to standardized data preprocessing. To mitigate the impact of missing data, data were supplemented by contacting liaison officers from Blood Services. Frequency and composition rates were used to describe the data of total donation, as well as data on the different types of ADRs. The Chi-square test was used to analyze risk factors for Vasovagal reactions (VVRs) and inter-group comparisons of VVRs stratified by age (18–22; 23–29; 30–39; 40–49; 50–60), gender (female/male), donation history (first time/repeat), and blood donation volume (200 mL/300 mL/400 mL), type of collection site (fixed site/blood collection shelter/blood collection vehicle/others) and donor source (individual/social group/high school). The same stratified analysis was further conducted on blood donors who experienced syncope to determine the risk factors. R software (version 4.2.3, R Foundation for Statistical Computing, New Zealand) was employed for statistical analyses (13). The Odds ratio (OR) and its 95% confidence interval (95% CI) were calculated to compare the reported incidence of VVR with different characteristics, *p* < 0.05 was considered to be statistically significant, and all *p* values were tested using a bilateral test.

## Results

### Donor characteristics

A total of 8,575,731 blood donations, comprising 7,816,095 whole blood donations and 759,636 apheresis platelet donations, were registered at Blood Services during the study period. The general donor characteristics for the study population are listed in Table 1. The analysis of the characteristics of whole blood donors showed that, donors aged 30–39 (26.4%), 40–49 (23.2%), and 18–22 (22.7%) account for 72.3% of the total whole blood donors. Male donors (59.7%) outnumber female donors (40.3%). Repeat donors (54.9%) were more than first-time donors. The majority of donors contributed 400 mL of blood (63.3%). Blood collection from mobile blood donation vehicles was the most common (63.0%). Individual donors (63.0%) far exceed those from social and university groups.

**Table 1:** Demographic characteristics of blood donors.

Donation type	Characteristics		Donations				
			2020	2021	2022	Total	Total %
Whole blood	Age (years)	18–22	490,583	711,207	572,402	1,774,192	22.7
		22–29	345,670	372,435	457,625	1,175,730	15.0
		30–39	583,381	649,116	831,804	2,064,301	26.4
		40–49	517,736	572,324	724,547	1,814,607	23.2
		50–60	289,372	299,660	398,233	987,265	12.7
	Gender	Male	1,363,106	1,531,778	1,771,865	4,666,749	59.7
		Female	863,636	1,072,964	1,212,746	3,149,346	40.3
	Donation history	First time	1,031,472	1,221,088	1,272,470	3,525,030	45.1
		Repeat	1,195,315	1,383,654	1,712,141	4,291,110	54.9
	Volume (mL)	200	347,265	394,648	403,436	1,145,349	14.8
		300	409,960	591,120	684,910	1,685,990	21.9
		400	1,369,159	1,616,326	1,896,265	4,881,750	63.3
	Type of collection site	Fixed site	49,760	52,414	124,826	227,000	2.9
		Blood collection shelter	734,185	739,124	863,306	2,336,615	29.9
		Blood collection vehicle	1,361,157	1,783,441	1,774,459	4,919,057	63.0
		Others	72,785	30,001	222,020	324,806	4.2
	Donor source	Individual	1,390,683	1,634,769	1,885,937	4,911,389	63.0
		Social group	598,713	565,104	798,986	1,962,803	25.2
		High school	214,728	405,042	299,688	919,458	11.8
Apheresis platelet	Age (years)	18–22	16,634	35,032	35,666	87,332	11.5
		22–29	26,933	38,684	51,938	117,555	15.5
		30–39	52,703	74,678	93,394	220,775	29.0
		40–49	50,436	64,818	84,422	199,676	26.3
		50–60	29,574	44,535	60,189	134,298	17.7
	Gender	Male	136,618	200,311	253,691	590,620	77.7
		Female	40,146	57,436	71,918	169,500	22.3
	Donation history	First-time	23,378	21,835	26,369	71,582	9.4
		Repeat	153,386	235,912	299,240	688,538	90.6
	Dose	Single	67,393	84,563	95,946	247,902	32.6
		Double	110,137	173,209	229,663	513,009	67.4
	Type of collection site	Fixed site	127,019	159,784	236,208	523,011	68.7
		Blood collection shelter	50,123	85,460	88,079	223,662	29.4
		Blood collection vehicle	943	12,580	1,260	14,783	1.9
		Others	8	0	62	70	0.0
	Donor source	Individual	165,909	239,680	310,856	716,445	94.7
		Social group	5,604	8,878	12,706	27,188	4.1
		High school	1,669	9,247	2,047	12,963	2.0
		Others	0	3	0	3	0.0

Among apheresis platelet donors, those aged 30–39 (29.0%) and 40–49 (26.3%) accounted for 55.3% of the total apheresis platelet donors. Male donors (77.7%) were more than female donors (22.3%). Repeat donors (90.6%) were significantly more than first-time donors. The majority of donors contributed two therapeutic doses of platelets (67.4%). Donations were mainly made at fixed donation sites (68.7%). At the same time, individual donors were the main source (94.69%).

### Classification of ADRs

A total of 32,523 donations with ADRs were reported in Blood Services, among which 191 cases experienced two types of ADRs during the same donation from 2020 to 2022. Of these ADRs, 30,933 ADRs were related to whole blood donors, and the remaining 1,781 ADRs were associated with apheresis platelet donors. VVRs (31,498; 96.32%) were the most prevalent type of ADRs in blood donors, followed by hematoma (792; 2.56%). Next, the three VVR subclusters were subdivided according to the pre-defined grouping criteria. Among the 31,498 donors who experienced VVRs, loss of consciousness (LOC) was reported in 1,120 (3.36%). When ADRs could not be classified into the existing categories, they will be classified under the “Other” category. For example, a blood donor has a fever after donating blood (Table 2).

**Table 2:** Frequency of ADRs of whole blood and apheresis platelet donors.

Donation type	Type of ADRs		Frequency				
			2020	2021	2022	Total	Total %
Whole blood	Localized	Haematoma (Bruise)	44	145	101	290	0.94
		Arterial puncture	0	2	0	2	0.01
		Delayed bleeding	2	7	3	12	0.04
		Nerve injury	1	9	0	10	0.03
		Painful arm	11	75	50	136	0.44
		Allergy (local)	0	0	3	3	0.01
	Systemic (VVRs)	With LOC	264	334	461	1,059	3.42
		Without LOC	5,835	11,465	12,100	29,400	95.04
		With injury	12	18	48	78	0.25
		Without injury	6,087	11,781	12,513	30,381	98.22
		On collection facility	6,037	11,702	12,390	30,129	98.92
		Outside collection facility	62	97	171	330	1.08
	Others		0	17	4	21	0.07
Apheresis platelet	Localized	Haematoma (Bruise)	6	283	213	502	31.53
		Painful arm	2	5	0	7	0.44
		Allergy (local)	1	1	0	2	0.13
	Systemic (VVRs)	With LOC	18	14	29	61	3.83
		Without LOC	154	356	468	978	61.43
		With injury	1	0	3	4	0.25
		Without injury	171	370	494	1,035	65.01
		On collection facility	170	368	495	1,033	64.89
		Outside collection facility	2	2	2	6	0.38
	Related to apheresis	Citrate reaction	49	82	89	220	13.82
		Generalized allergic reaction	1	1	1	3	0.19
	Others		0	4	4	8	0.50

### Incidence of ADRs

The reported ADR incidence was 12.577% (24,678/1,962,174) between 2020 and 2022. In whole-blood donors, the incidence of ADRs was 13.248% (23,655/1,785,628), which was significantly higher than the 6.015% (1,062/176,546) observed in apheresis platelet donors (*p* = 0.0013). The frequency and incidence of each ADR category are detailed in Table 3.

**Table 3:** Frequency and incidence of ADRs of the top 20% blood establishments.

Donation type	Type of ADRs	Frequency *n* (%)	Total donations	Incidence rate: per 1,000 donations (95% CI)	Incidence rate: 1 per × donations
Whole blood	Systemic (VVRs)	23,445 (99.11)	1,785,628	13.130 (12.963–13.297)	76
	Haematoma (Bruise)	147 (0.62)		0.082 (0.069–0.096)	12,195
	Painful arm	44 (0.19)		0.025 (0.017–0.032)	40,000
	Nerve injury	8 (0.03)		0.004 (0.001–0.008)	250,000
	Delayed bleeding	6 (0.03)		0.0003 (0.001–0.006)	333,333
	Arterial puncture	1 (0.01)		0.001 (0.000–0.002)	1,000,000
	Other	5 (0.02)		0.003 (0.000–0.005)	333,333
	Total ADRs	23,616 (100.00)		13.248 (13.080–13.416)	75
Apheresis platelet	Haematoma (Bruise)	478 (45.00)	176,546	2.708 (2.518–3.008)	369
	Systemic (VVRs)	458 (43.14)		2.594 (2.357–2.832)	386
	Citrate reaction	110 (10.36)		0.623 (0.507–0.739)	1,605
	Painful arm	5 (0.47)		0.028 (0.003–0.053)	35,714
	Generalized allergic reaction	2 (0.19)		0.011 (0.000–0.027)	90,909
	Allergy (local)	1 (0.09)		0.006 (0.000–0.017)	166,667
	Other	8 (0.75)		0.045 (0.014–0.077)	22,222
	Total ADRs	1,062 (100.00)		6.072 (5.710–6.434)	165

### Risk factors for VVRs

**Figure 1:**
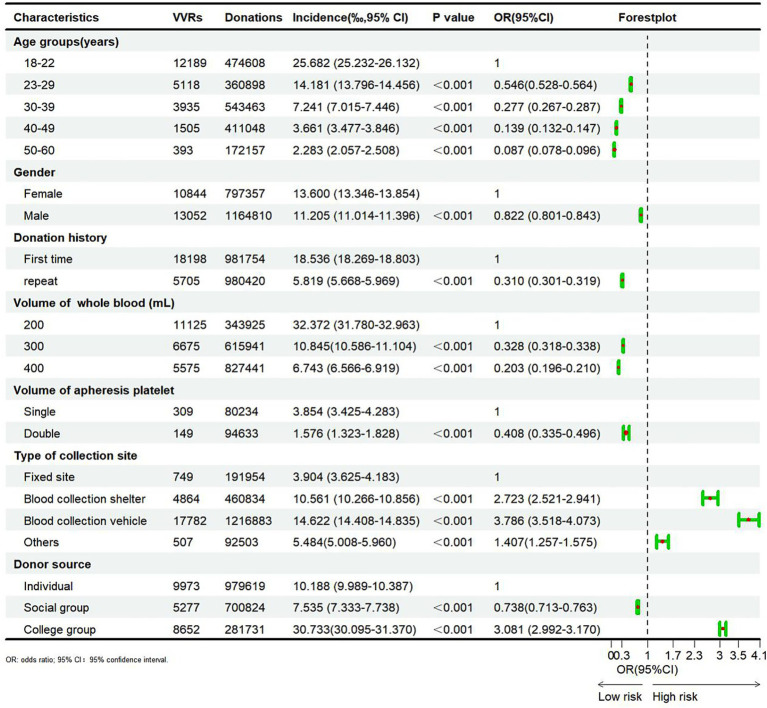
Results of risk factors analysis of blood donors experienced VVRs.

The results of the chi-square test regarding risk factors for VVRs are illustrated in Figure 1. Of note, the incidence of VVRs decreased with advancing age (*p* < 0.01). It was highest in the 18–22 (25.682%) age group and lowest in the 50–60 age group (2.283%). Compared with the 18–22 age group, the odds ratios (OR) for VVR were 0.546, 0.277, 0.139, and 0.087 in the 22–29, 30–39, 40–49, and 50–60 age groups, respectively.

The incidence of VVRs in male blood donors (11.205%) was significantly lower than that in females (13.600%) (*p* < 0.01, OR = 0.822). Likewise, the incidence of VVRs was significantly lower than in repeat donors (5.819%) compared to first-time donors (18.536%) (*p* < 0.01, OR = 0.310).

The incidence of VVRs decreased with the increasing blood donation volume. Among whole blood donors, the incidence of VVRs (32.372%) was significantly higher among those donating 200 mL compared to those donating 300 mL (10.845%) and 400 mL (6.743%) (*p* < 0.01). The same trend was observed for apheresis platelet donors. Additionally, the incidence of VVRs was significantly lower at fixed blood donation sites compared to other blood donation sites (*p* < 0.01). Compared with individual blood donors (10.188%), the incidence of VVRs was significantly lower in social group donors (7.535%) and significantly higher in college group donors (30.733%) (*p* < 0.01, OR = 0.738 and 3.081).

### Risk factors for syncope

**Figure 2:**
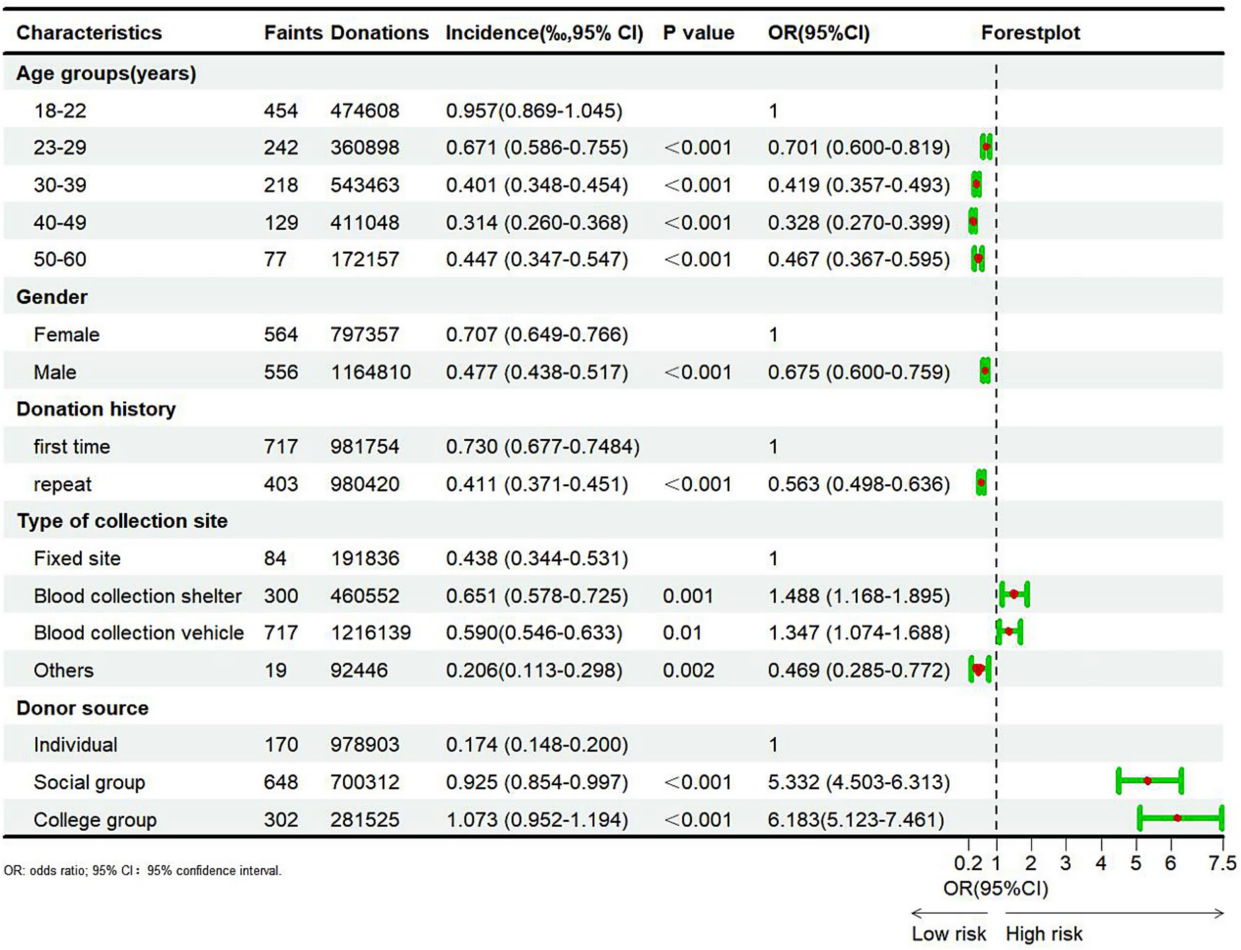
Results of risk factors analysis of blood donors experienced syncope.

A total of 1,120 VVRs with syncope were reported across sentinel Blood Services from 2020 to 2022, accounting for 3.56% of all total VVR cases (1,120/31,498). The results of the chi-square and Cochran-Armitage tests regarding risk factors for syncope are listed in Figure 2. Significant correlations were noted between the incidence of syncope and age, gender, donation history, type of collection site, and donor source (*p* < 0.05). The incidence of syncope decreased with advancing age (*p* < 0.001). Specifically, the incidence of syncope was highest in the 18–22 (0.957%) age group and lowest in the 40–49 (0.314%) age group. Compared with the 18–22 age group, the odds ratios (OR) for syncope in the 22–29, 30–39, 40–49, and 50–60 age groups were 0.701, 0. 419, 0.328, and 0.467, respectively. Furthermore, the incidence of syncope was significantly lower in male donors (0.477%) compared to female donors (0.707%) (*p* < 0.001, OR = 0.675). Similarly, the incidence of syncope was significantly lower in repeat donors (0.411%) compared to first-time donors (0.730%) (*p* < 0.001, OR = 0.563). Differences in the incidence of syncope across blood donation sites were statistically significant, with its incidence being lower in fixed blood donation sites compared to blood donation vehicles or shelters. Finally, the incidence of syncope in social group donors (0.925%) and university donors (1.073%) was significantly higher compared to individual donors (0.174%) (*p* < 0.001, OR = 5.332 and 6.183).

## Discussion

VVRs accounted for 98.47% and 58.34% of all ADRs in whole blood and apheresis platelet donors from 2020 to 2022 in China, respectively. It is worthwhile emphasizing that the rate of VVRs in whole blood donors was higher than that reported in Italy from 2016 to 2019 (93.3%) (14), IHN (83%) (15), and the 2023 European Commission SARE report (82%) (16). The rate of VVRs among apheresis platelet donors was lower than that recorded in Italy (70.3%) from 2016 to 2019 and comparable with that of IHN (57%) and the 2023 European Commission SARE report (77%). Among whole blood donors, local ADRs such as hematoma following blood donation accounted for only 0.94% of all ADRs, which was markedly lower than the 18.69% reported by IHN (8), given that whole blood donors often discover hematomas after leaving the blood collection site and may not inform Blood Services of the adverse reaction. However, the longer process of apheresis platelet collection increases the likelihood of on-site diagnosis, and the proportion of hematomas recorded in apheresis platelet donors was higher at 31.53%.

According to earlier studies, Blood Services did not always collect and report all ADR data (10). To mitigate this bias and reflect the real rate of ADRs, data from Blood Services with a reported incidence of over 5% were used to calculate the incidence of ADRs to capture a more accurate representation of blood donor safety in China. These Blood Services accounted for 20% of the total number of participating Blood Services, thereby presenting a more reliable basis for assessing adverse associated with ADRs following blood donation in China.

The total reported incidence of whole blood ADRs was 13.248%. The result was consistent with the results of a meta-analysis published in 2021 that investigated the incidence of ADRs in Chinese whole blood donors across 36 studies, revealing a combined reported incidence of 13.6% (9). Compared with whole blood donors, the overall reported incidence of ADRs in platelet donors was lower (6.072%). These results are lower than those reported in a meta-analysis examining the incidence of ADRs in platelet apheresis donors published in 2024 (including 91 articles), which reported a combined incidence of 2.65% in China (17). The incidence of ADRs reported by apheresis platelet donors is strongly correlated with awareness and reporting practices. The results of an analysis from Denmark’s donor hemovigilance system demonstrated that the reported incidence of citrate reaction ranges from 0% to 45.8% (18), attributable to differences in information systems, methods of detection, passive reception, reporting personnel, and the possibility of under reporting based on donor feedback.

Considering that VVRs were the most prevalent ADRs, risk factors of VVRs were explored among Chinese blood donors, focusing on age, gender, blood donation history, blood donation site, and donor source. In terms of age groups, the risk of VVRs was highest in blood donors aged 18–22 years and decreased with increasing age, consistent with the AABB reports between 2012 and 2017 (19). The 18–22 age group largely consisted of college students, mostly first-time blood donors. Their limited knowledge of blood donation and social adaptability, could easily lead to mental tension during blood donation, resulting in VVRs (20). Regarding gender, female blood donors were at a higher risk for VVRs herein, attributable to their lower body weight (21). Moreover, first-time blood donors were approximately three times more likely to experience VVRs compared to repeat blood donors, highlighting the need for education and support for first-time blood donors. Interestingly, contrary to our expectations, the incidence of VVRs was highest in blood donors who donated 200 mL of whole blood. The incidence of VVRs in apheresis platelet donors was also higher in those who donated single unit compared with those who donated double units, indicating that in China, donations of up to 400 mL of whole blood and two treatment doses of platelets are not significant risk factors for inducing VVRs (22). Of course, this is related to the conditions of blood donors, first-time, young, female blood donors often choose to donate smaller volume of blood. As many countries do not practice whole blood volume diversity, this point would merit further investigation. The incidence of VVRs also varied across blood donation sites. Specifically, the incidence of VVRs was highest in blood donation vehicles, followed by the fixed blood donation house, whilst the incidence of VVRs was lowest in blood donation stations. It is worth noting that the confined space and higher personnel density in blood donation vehicles, as well as the elevated noise levels and poor air circulation, may contribute to discomfort among donors, thus increasing the incidence of VVRs (23). Blood donors in China can originate from individuals, social groups, and college groups. The incidence of VVRs in college groups was substantially higher than that of blood donors from other sources due to their age and blood donation history (24, 25).

Blood donation-related vasovagal syncope may lead to severe complications such as fractures, dental injuries, and even death (26). Among the 1,120 cases of syncope recorded in this study, 677 cases occurred during blood collection, whereas 371 cases occurred during the rest period after blood donation, highlighting the need to monitor donors after blood donation and before allowing them to leave the blood collection site. Herein, risk factors for syncope were investigated. Age groups at risk of syncope were similar to those observed with VVRs. Nonetheless, the risk of syncope in the 50–60 age group was higher than that in the 30–49 age group, with the majority of blood donors in China being repeat blood donors, indicating that the risk of syncope after blood donation in the older population warrants increased attention. Although previous studies have established that repeat blood donors remain at risk for syncope (27), the results of this study demonstrated that repeat blood donors had a significantly lower risk of syncope compared to first-time blood donors. Inconsistent with the findings for VVRs, the risk of syncope among social group and college group donors was 5–6 times higher than that of individual donors, which may be related to the group dynamics associated with VVRs (28).

Some limitations of this study cannot be overlooked. Firstly, given the lack of connectivity between the blood donor management information system among participating blood stations, detailed information on all blood donors was not obtained. Therefore, the multivariate analysis method was not employed to examine risk factors for ADRs, and only univariate analyses were conducted. Secondly, at the beginning of this project, a new evaluation tool for rating the severity rating of ADRs had not been published; consequently, it was not used for analysis. Finally, rare, severe, long-term ADRs were not explored and may be addressed in future studies using the BEST methods in multi-center studies (29).

In conclusion, to the best of our knowledge, this study represents the first large-scale analysis of national donor hemovigilance, providing an initial reflection of the current state of blood donor safety in China. The incidence of ADRs was low and consistent with the rates reported by other national haemovigilance systems. Among donors experiencing VVRs and syncope, the risk was higher in first-time females, those donating on blood donation mobiles, and college groups.

## Data Availability

The raw data supporting the conclusions of this article will be made available by the authors, without undue reservation.

## Footnotes

^1^ https://hv.cqsbt.org.cn
