# Supplementary material for: Profile and risk factors of blood donors who experienced adverse reactions: a cross-sectional study on donor hemovigilance data reported to the national network from 2020 to 2022 in China
Source: Front Public Health. 2025 Nov 26;13:1567370. doi: 10.3389/fpubh.2025.1567370 (PMC12689900; doi:10.3389/fpubh.2025.1567370)
Supplement: Supplementary file 2 [file Data_Sheet_2.pdf]

Summary Table of Adverse Reactions to Blood Donation

No.	Blood collection date	Gender	Age	Weight(kg)	Height(cm)	Types of blood donation	Volume	Donation history	Type of collection site	Donor source	Signs and symptoms	Reaction type	Imputability	Follow-up information
